# iSuc-PseAAC: predicting lysine succinylation in proteins by incorporating peptide position-specific propensity

**DOI:** 10.1038/srep10184

**Published:** 2015-06-18

**Authors:** Yan Xu, Ya-Xin Ding, Jun Ding, Ya-Hui Lei, Ling-Yun Wu, Nai-Yang Deng

**Affiliations:** 1 Department of Information and Computer Science, University of Science and Technology Beijing, Beijing 100083, China; 2 Institute of Applied Mathematics, Academy of Mathematics and Systems Science, Chinese Academy of Sciences, Beijing 100190, China; 3 College of Science, China Agricultural University, Beijing 100083, China

## Abstract

Lysine succinylation in protein is one type of post-translational modifications (PTMs). Succinylation is associated with some diseases and succinylated sites data just has been found in recent years in experiments. It is highly desired to develop computational methods to identify the candidate proteins and their sites. In view of this, a new predictor called iSuc-PseAAC was proposed by incorporating the peptide position-specific propensity into the general form of pseudo amino acid composition. The accuracy is 79.94%, sensitivity 51.07%, specificity 89.42% and MCC 0.431 in leave-one-out cross validation with support vector machine algorithm. It demonstrated by rigorous leave-one-out on stringent benchmark dataset that the new predictor is quite promising and may become a useful high throughput tool in this area. Meanwhile a user-friendly web-server for iSuc-PseAAC is accessible at http://app.aporc.org/iSuc-PseAAC/ . Users can easily obtain their desired results without the need to understand the complicated mathematical equations presented in this paper just for its integrity.

Protein post-translational modification (PTM) is one of the most efficient biological mechanisms for expanding the genetic code and for regulating cellular physiology^1^. Lysine succinylation is one type of PTMs. The succinyllysine residue was initially identified by mass spectrometry and protein sequence alignment. The research further showed that lysine succinylation is evolutionarily conserved and responds to different physiological conditions^2^. Park *et al.*^3^ identified 2565 succinylation sites on 779 proteins in 2013. They revealed potential impacts of lysine succinylation on enzymes involved in mitochondrial metabolism such as, amino acid degradation, the tricarboxylic acid cycle (TCA) and falty acid metabolism. SIRT5 has been found as the known enzyme to catalyze lysine desuccinylation^3^,^4^. Lysine succinylation is also present on histones, suggesting possible roles in regulating chromatin structures and functions^5^. Therefore, identifying the succinylated sites in proteins may provide useful information for biomedical research.

Identification of succinylation residues with experiments was mainly by means of mass spectrometry, which was expensive and laborious. Facing the avalanche of protein sequences generated in the post genomic age, it is a supplementary way to develop computational methods for timely and effectively identifying the succinylation residues in proteins.

There are not computational methods to identify lysine succinylation sites. The present study was devoted to develop a new predictor for identifying lysine succinylation in proteins incorporating the peptide position-specific propensity into the general form of pseudo amino acid composition. According to a comprehensive review^6^, to develop a really useful predictor for a protein system, we usually need to consider the following procedures: (a) select or construct a valid benchmark dataset to train and test the predictor; (b) represent the protein or peptide samples with an effective formulation that can truly reflect their intrinsic correlation with the target to be predicted; (c) introduce or develop a powerful algorithm or operation engine to conduct the prediction; (d) properly perform cross-validation tests to objectively evaluate the anticipated prediction accuracy; (e) establish a user-friendly web-server for the predictor that is accessible to the public.

## Methods

### Benchmark Dataset

In this study the benchmark dataset was derived from the CPLM^7^ which was a protein lysine modification database. There are 2521 lysine succinylation sites and 24128 non-succinylation sites in 896 unique proteins. The corresponding protein sequences were derived from Uniprot database^8^. For facilitating description later, let us adopt the Chou’s peptide formulation which was used for signal peptide cleavage sites^9^, and S-Nitrosylation site prediction^10^. According to Chou’s scheme, a peptide with lysine (K) located at its center can be expressed as


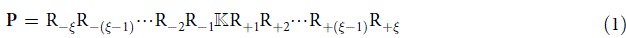

where the subscript 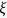 is an integer, 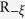 represents the 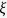-th downstream amino acid residue from the center, 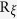 the 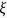-th upstream amino acid residue, and so forth. A peptide 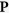 is classified into the following categories:


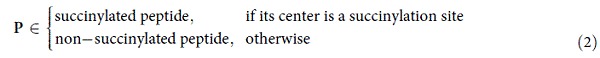


Thus, the benchmark dataset can be formulated as


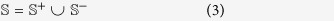


where 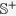 contains the samples for the succinylated peptides only, 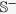 contains the non-succinylated peptides only (cf. Eq.2).

The parameter 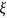 in peptides was 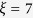 after some preliminary trials and the sample extracted from proteins in this study was a 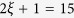 tuple peptide. If the upstream or downstream in a peptide sample was less than 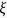, the lacking residues were filled with the dummy code X. The experimental results would be overestimated if the benchmark dataset contained homology peptides. Those peptides that had 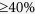 pairwise sequence identity to any other were rigorously excluded from the benchmark datasets.

Finally, we obtained the benchmark dataset 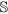 containing 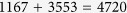 peptide samples in Table 1, of which 1167 were succinylated peptides belonging to the positive subset 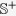, and 3553 were non-succinylated peptides belonging to the negative subset 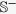. The peptide fragments as well as their succinylation or non-succinylation sites in proteins are given in the Supplementary Materials S1 and S2 for 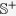 and 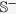, respectively.

### Feature Vector Construction

The peptides need to convert into effective mathematical expression (feature construction) which could reflect intrinsic correlation with the desired target in predicting the PTMs. The protein sequences are the most and important information to construct features. According to the review^6^, the general form for a protein or peptide 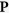 can be formulated by


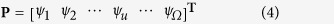


where 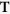 is the transpose operator and 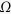 is an integer to reflect the vector’s dimension. The value of 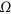 as well as the components 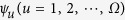 in Eq.4 will depend on how to extract the desired information from the protein or peptide sequences. Below, let us describe how to extract the useful information from the benchmark dataset 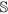 to define the peptide samples via Eq.4.

A peptide 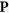 in Eq.1 can be simplified to a more convenient form given by


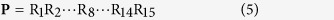


where 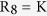, and 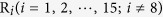 can be any of the 20 native amino acids or the dummy code X. We use the numerical codes 1, 2, 3, 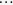, 20 to represent the 20 native amino acids according to the alphabetic order of their single letter code, and use 21 to represent the dummy amino acid X. A “Position Specific Amino Acid Propensity” (PSAAP) matrix 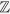
10,11 was introduced according to the benchmark dataset 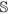.


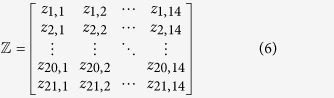


where the element
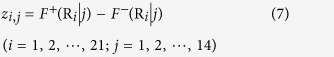

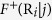
 is the occurrence frequency of the 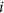-th amino acid (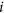 = 1, 2, 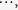 21) in the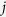-th column in the positive benchmark dataset 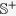 while 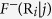 is the corresponding occurrence frequency but derived from the negative benchmark dataset 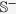. We deleted the center amino acid K as it was the same in positive and negative peptides (samples), respectively. Thus, the components in Eq.4 can be uniquely defined by


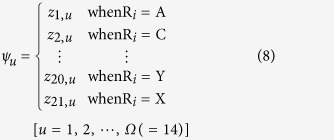


### Prediction Algorithm

Support vector machine (SVM) is one of the most widely used machine learning algorithms in bioinformatics. The decision rule g(x) was obtained by solving a convex quadratic programming with kernel function. In this work, the kernel function was RBF (Radial Basis Function) kernel with parameter 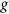 = 0.005. In order to obtain the probability output from SVM, i.e. the probability of that unlabeled input x belongs to a certain class, P(y = 1|x), a logistic model was built to map the output g(x) of the SVM into estimated probabilities^12^.


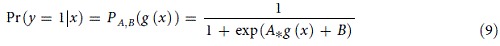


Parameter *A* and *B* can be obtained by solving the following model


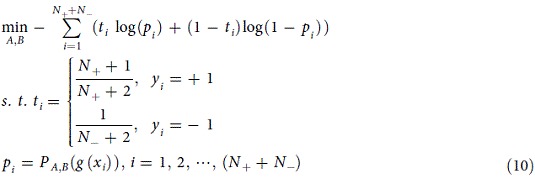


where 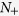 and 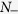 represent the number of 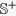 and 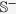 during training process, respectively.

For a query peptide 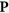 as formulated by Eq.4, suppose 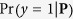 is its probability to the succinylated peptides. Thus, the prediction rule for the query peptide 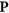 can be formulated as


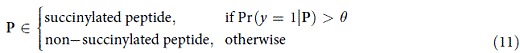


The cutoff value θ is 0.35 for balancing the true positive and negative rate, unless an additional introduction is attached. The SVM algorithm is implemented by LIBSVM, a public and widely used SVM library.

**Figure 1:**
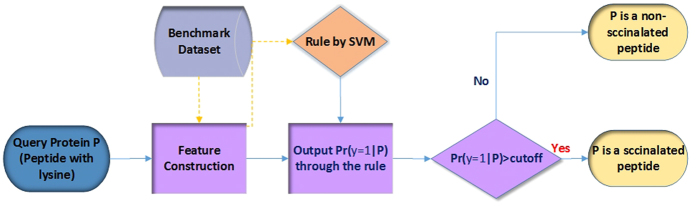
A flowchart of the iSuc-PseAAC predictor.

The predictor established via the above procedures is called **iSuc-PseAAC**, where “i” stands for the 1^st^ character of “identify”, “Suc” for “succinylation”, and “PseAAC” for that the general form of pseudo amino acid composition was used to formulate the peptide sequences. A flowchart of the predictor was given in Fig. 1 to illustrate how **iSuc-PseAAC** worked during the process of prediction.

### Four metrics for measuring prediction quality

To measure the performance of the predictor **iSuc-PseAAC**, four usual metrics were adopted as in^10^,^13^,^14^,^15^,^16^ and they are defined as


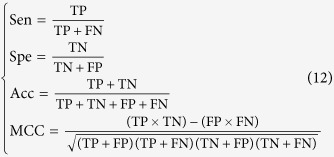


where TP (true positive) denotes the number of succinylated peptides correctly predicted, TN (true negative) the numbers non-succinylated peptides correctly predicted, FP (false positive) the non-succinylated incorrectly predicted as the succinylated peptides, and FN (false negative) the succinylated peptides incorrectly predicted as the non-succinylated peptides. Sen, Spe, Acc, and MCC are the sensitivity, specificity, accuracy and the Mathew’s correlation coefficient^17^, respectively. The ROC curve (receiver operating characteristic curve) which shows the trade-off between sensitivity and specificity is also been examined. AUC (area under the curve) is also another indicator in practical application. It is instructive to point out that the metrics as defined in Eqs.12 are valid for single-label systems; for multi-label systems a set of more complicated metrics should be used as given in^18^.

## Results and Discussion

### Leave-one-out Cross Validation

The cross validation methods are often used to examine the quality of a predictor and its effectiveness in PTMs. The independent dataset test, subsampling or K-fold (such as 6-fold, 8-fold, or 10-fold) cross validation test and leave-one-out (LOO) test are the most cross validations. The K-fold cross validation was used for its less computational time and often been performed many times for different subsampling combinations followed by averaging their outcomes as done by investigators for PTM site predictions^19^,^20^,^21^,^22^. The LOO test is the least arbitrary that can always yield a unique result for a given benchmark dataset. Therefore, it has been widely recognized and increasingly utilized to examine the quality of various predictors (see, e.g.,^18^,^23^,^24^,^25^). Accordingly, in this study the LOO and K-fold cross validation were adopted to evaluate the accuracy of the current predictor. The 10-fold, 8-fold and 6-fold cross validations have been executed for 30 times to avoid the bias. Their results obtained by iSuc-PseAAC on the benchmark dataset were listed in Table 2.

**Figure 2:**
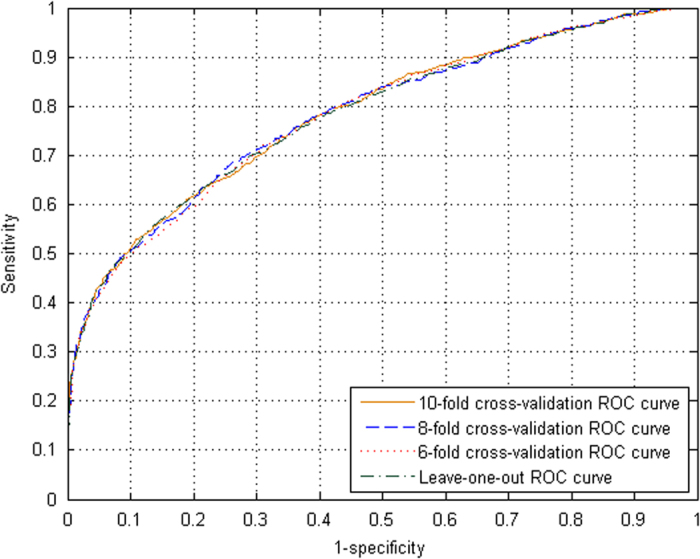
The ROC curves for the LOO test and 6-, 8-, 10-fold cross-validations.

As we can see from Table 2, the overall accuracies for the lysine succinylation was (80.02 ± 0.27)% and its sensitivity (50.65 ± 0.63)%, specificity (89.67 ± 0.27)%, MCC (0.432 ± 0.007) and the AUC (0.782 ± 0.003) in 10-fold cross validation. The AUC were (0.782 ± 0.002) and (0.781 ± 0.002) in 8-fold and 6-fold cross validation, respectively. In LOO test the accuracy was 79.94%, sensitivity 51.07%, specificity 89.42% and AUC 0.782. The ROC curves in Fig.2 were intensive which illustrated the robust of the predictor iSuc-PseAAC. All these results in cross validations and LOO test were approximate. (in Table 2 and Fig.2).

**Figure 3:**
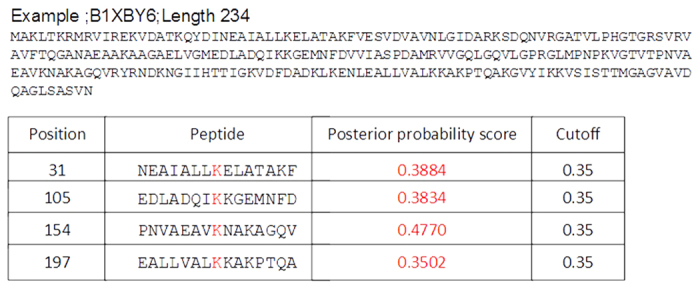
The predicted results of the predictor iSuc-PseAAC.

As pointed out in^26^, and emphasized in a series of recent publication (see, e.g.,^27^,^28^), another key in developing a practically useful prediction method is to establish a user-friendly and publicly accessible web-server. In view of this, the web server for **iSuc-PseAAC** has been established that can be freely accessible at http://app.aporc.org/iSuc-PseAAC/. Users can easily get the desired result by using **iSuc-PseAAC** without the need to follow the complicated mathematical equations presented in this paper. Either type or copy/paste the query protein sequences into the input box or upload your input files. The protein sequences should be in FASTA format. Click on the Submit button to see the predicted results in Fig.3. For example, protein B1XBY6 has lysine succinylation 105, 154, 186 and 197 sites, and the predictor iSuc-PseAAC has successfully predicted 31, 105, 154 and 197 sites. Protein E9Q5L3 has three succinylation sites (70, 278 and 284) and iSuc-PseAAC has successfully predicted 278 and 284 sites. Click on the Data button to download the benchmark dataset.

## Additional Information

**How to cite this article**: Xu, Y. *et al.* iSuc-PseAAC: predicting lysine succinylation in proteins by incorporating peptide position-specific propensity. *Sci. Rep.* doi: 10.1038/srep10184 (2015).

## Supplementary Material

Supplementary Material S1

Supplementary Material S2

## Figures and Tables

**Table 1:** The number of positive and negative peptides in the benchmark dataset 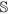 .

_No._	_Positive_	_Negative_
Homologous	_2521_	24128
Non-redundancy	_1167_	3553

**Table 2:** The 10-fold, 8-fold and 6-fold cross-validation results by the predictor on the benchmark dataset 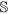 .

_**Cross-validation**_	_**Sen (%)**_	_**Spe (%)**_	_**Acc (%)**_	_**AUC**_	_**MCC**_
10-fold	50.65 ± 0.63	89.67 ± 0.27	80.02 ± 0.27	0.782 ± 0.003	0.432 ± 0.007
8-fold	50.25 ± 0.90	89.65 ± 0.34	79.91 ± 0.27	0.782 ± 0.002	0.428 ± 0.007
6-fold	49.95 ± 0.62	89.70 ± 0.35	79.87 ± 0.35	0.781 ± 0.002	0.426 ± 0.009
LOO	51.07	89.42	79.94	0.782	0.431

The experiments have been executed 30 times for every cross-validation and the results were the mean 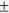 standard variation.
